# Bladder Preservation Therapy in Muscle-Invasive Bladder Cancer: Current Evidence and Future Directions

**DOI:** 10.3390/jcm15135101

**Published:** 2026-06-30

**Authors:** Patrick P. Carriere, Comron J. Hassanzadeh

**Affiliations:** Division of Radiation Oncology, Department of Genitourinary Radiation Oncology, The University of Texas MD Anderson Cancer Center, 1515 Holcombe Blvd, Houston, TX 77030, USA; ppcarriere@mdanderson.org

**Keywords:** muscle-invasive bladder cancer, bladder preservation, trimodality therapy, radiation therapy

## Abstract

Bladder preservation has emerged as an established treatment option for selected patients with muscle-invasive bladder cancer (MIBC), offering durable oncologic control with the potential to maintain native bladder function and quality of life. Over the past several decades, prospective trials and large institutional experiences have refined trimodality therapy (TMT)—maximal transurethral resection followed by definitive radiation therapy with concurrent radiosensitizing systemic therapy—and clarified principles of patient selection, treatment delivery, surveillance, and salvage. Randomized evidence supports combined-modality therapy as the backbone of bladder preservation, and contemporary comparative analyses suggest outcomes comparable to radical cystectomy in appropriately selected populations. This review synthesizes the clinical foundations of bladder preservation, including radiobiologic considerations, advances in radiation technique, and patterns of recurrence following TMT. We discuss outcomes in higher-risk populations, including locally advanced and node-positive disease, and examine the evolving integration of systemic therapies. The emergence of immune checkpoint inhibitors and antibody–drug conjugates in urothelial carcinoma has reshaped the systemic treatment landscape and raises important questions regarding patient selection, sequencing, and the potential expansion of organ-preserving strategies. Finally, we outline future directions—including response-adaptive approaches, advances in image-guided and adaptive radiotherapy, and ctDNA-enabled risk stratification—while emphasizing the need for prospective validation and multidisciplinary collaboration to refine and optimize bladder-preserving care.

## 1. Introduction

Muscle-invasive bladder cancer (MIBC), the most aggressive localized presentation of urothelial carcinoma (UC), has historically been managed with radical cystectomy and pelvic lymph node dissection. Although cystectomy remains a curative standard, it carries meaningful perioperative morbidity and long-term functional consequences related to urinary diversion, sexual health, and overall quality of life. In addition, a substantial proportion of patients are medically ineligible for major surgery, and others prioritize organ preservation when oncologically appropriate. These considerations have driven sustained interest in bladder-preserving strategies over the past several decades.

Trimodality therapy (TMT)—maximally safe transurethral resection of bladder tumor (TURBT) followed by definitive radiation therapy (RT) with concurrent radiosensitizing systemic therapy—has emerged as the most established bladder preservation approach. Prospective randomized trials and mature institutional series have demonstrated that in addition to preserving bladder function, TMT can achieve durable local control and favorable long-term survival outcomes similar to those reported for radical cystectomy in carefully selected patients [1,2,3,4]. However, there is currently an absence of randomized trials directly comparing TMT with radical cystectomy and retrospective data can be limited by selection bias. The phase III BC2001 trial established the benefit of concurrent chemoradiation over RT alone, solidifying combined-modality therapy as the backbone of bladder preservation [1]. Reflecting this evidence base, contemporary clinical guidelines now recognize bladder preservation as a standard option for appropriately selected patients with MIBC [5,6].

The therapeutic landscape of MIBC, however, is rapidly evolving. Historically, bladder preservation was largely applied to organ-confined disease, with more limited data in locally advanced or node-positive presentations. Advances in RT delivery, improved multidisciplinary coordination, and the expansion of systemic therapy options have prompted reconsideration of these boundaries. In particular, the emergence of immune checkpoint inhibitors and antibody–drug conjugates has altered the management of locally advanced and metastatic MIBC and extended survival in metastatic settings. These developments raise important questions regarding patient selection, treatment sequencing, and whether improved systemic disease control may broaden candidacy for organ-preserving strategies in those with excellent systemic response with an intact bladder.

As outcomes improve in advanced UC, patterns of recurrence following bladder preservation and the management of limited metastatic or oligoprogressive disease have become increasingly relevant. Bladder preservation should therefore be viewed not as a static alternative to surgery, but as a dynamic component of modern multimodality care for MIBC.

In this review, we synthesize the contemporary evidence supporting bladder preservation in MIBC, including the biologic rationale for definitive chemoradiation, the clinical foundations of TMT, patient selection principles, outcomes in higher-risk disease subsets, integration of evolving systemic therapies, patterns of failure and salvage, and emerging technologies that may refine risk-adapted management. Our aim is to provide a balanced and critical appraisal of bladder preservation as a mature yet evolving strategy within the broader treatment landscape of MIBC.

## 2. Biological and Clinical Rationale for Bladder Preservation

The rationale for bladder preservation in MIBC is grounded in the principle that durable locoregional control can meaningfully influence both survival and symptom burden. Patterns-of-failure analyses consistently demonstrate that uncontrolled primary tumors contribute to morbidity and may facilitate metastatic dissemination, supporting aggressive local therapy as a core component of multimodality management [7]. In parallel, the evolution of the oligometastatic concept has reinforced the broader oncologic importance of maintaining durable local control, even within systemic treatment strategies [8].

UC exhibits intrinsic radiosensitivity, and the addition of concurrent systemic therapy enhances radiation response through complementary mechanisms, including augmentation of DNA damage and inhibition of repair pathways. Prospective randomized studies have demonstrated improved local control with radiosensitizing agents such as chemotherapy compared with RT alone, establishing combined-modality therapy as the biologic and clinical foundation of bladder preservation [3,9].

Bladder-specific radiobiologic considerations further strengthen the case for definitive RT. Tumor hypoxia, a well-recognized mediator of radioresistance, has been directly studied in MIBC. The phase III BCON trial demonstrated that hypoxia modification with carbogen and nicotinamide during RT significantly improved locoregional control and survival compared with RT alone [10]. These findings provide disease-specific evidence that the tumor microenvironment is not merely theoretical in bladder cancer, but clinically actionable. Importantly, they illustrate that radiation response in UC can be modulated—an observation that continues to inform contemporary efforts to integrate systemic therapies with definitive local treatment.

The sum of these biologic characteristics of UC provide a coherent mechanistic and clinical justification for bladder-preserving strategies in appropriately selected patients with MIBC.

## 3. Clinical Foundations of Bladder Preservation

### 3.1. Components and Sequencing of Trimodality Therapy

TMT represents the core bladder preservation strategy for MIBC, integrating maximal TURBT, definitive RT, and concurrent radiosensitizing systemic therapy. This coordinated approach seeks to eradicate visible disease, sterilize residual locoregional tumor burden, and address potential micrometastatic spread [1,2].

Maximally safe TURBT constitutes the first step of TMT and serves both staging and therapeutic purposes. Beyond improving pathologic and clinical assessment, aggressive endoscopic debulking reduces tumor burden prior to RT and identifies patients with high risk pathologic features favoring against TMT such as multifocal CIS and large tumors [11]. Multiple institutional series have demonstrated that completeness of TURBT correlates with higher complete response rates and improved long-term outcomes following CRT, underscoring the importance of urologic expertise within multidisciplinary bladder preservation programs [2,3,4,7,12].

RT provides the definitive local treatment component. Conventional fractionation schedules delivering approximately 64–66 Gy have historically been employed; however, contemporary practice increasingly incorporates moderately hypofractionated regimens delivering 55 Gy in 20 fractions supported by prospective and meta-analytic data demonstrating comparable tumor control and acceptable toxicity profiles [13]. Advances in RT delivery—including intensity-modulated RT (IMRT) and image guidance—have further improved conformality and normal tissue sparing [14,15], enhancing treatment safety without compromising oncologic efficacy.

Concurrent systemic therapy functions primarily as a radiosensitizer. The phase III BC2001 trial demonstrated superior invasive locoregional control with the addition of 5-fluorouracil and mitomycin C to RT compared with RT alone, establishing combined-modality therapy as the standard backbone of bladder preservation [3]. Alternative radiosensitizing regimens, including cisplatin- and gemcitabine-based approaches, provide options for patients with varying comorbidity profiles and renal function [16,17,18].

Cooperative group studies have further refined sequencing through response-adapted strategies incorporating interim cystoscopic assessment during or after induction therapy. Patients achieving complete clinical response may proceed with bladder preservation, whereas those with persistent invasive disease are directed toward early salvage cystectomy, preserving oncologic safety while maintaining the opportunity for organ preservation in responders [1,2]. However, the use of interim cystoscopy has fallen out of favor with modern TMT and instead cystoscopic evaluation is typically pursued after completion of TMT during the surveillance period. Long-term institutional experiences confirm the durability of this strategy and the feasibility of delayed surgical intervention when necessary [2].

In aggregate, the structure of TMT—maximally safe resection, definitive CRT, and disciplined response assessment—reflects a deliberate balance between oncologic rigor and functional preservation, forming the clinical scaffold upon which contemporary bladder-preserving strategies continue to evolve.

### 3.2. Patient Selection and Treatment Delivery

Patient selection remains central to successful bladder preservation and is increasingly recognized as existing along a clinical spectrum rather than a rigid binary framework (Table 1). Ideal candidates have traditionally included patients with cT2–T3 disease, unifocal tumors, absent or limited CIS, no hydronephrosis, preserved bladder function, and the ability to undergo maximal TURBT and rigorous surveillance [5,6]. At the same time, several factors historically viewed as contraindications may be better considered relative risk factors requiring individualized multidisciplinary assessment, including selected locally advanced or node-positive disease, limited CIS, unilateral hydronephrosis, or incomplete but substantial TURBT. Adequate renal function remains particularly relevant when cisplatin-based CRT is considered. While unselected metastatic disease is generally associated with poor candidacy, limited oligometastatic presentations may warrant individualized consideration.

Beyond disease-related factors, candidacy for TMT is also influenced by physiologic reserve, comorbidity burden, and ability to tolerate concurrent systemic therapy. Advanced age or comorbidity alone should not be considered contraindications to TMT, although frailty and competing medical illness may affect suitability for concurrent chemotherapy and warrant individualized treatment adaptation. Alternative radiosensitizing regimens may broaden eligibility in selected cisplatin-ineligible patients.

Although strict “ideal” candidacy criteria (e.g., unifocal disease, absence of extensive CIS or hydronephrosis, maximal TURBT, preserved bladder function) may apply to only a minority of patients with MIBC—often estimated at approximately 15–30% in highly selective historical frameworks [2,5,6]—contemporary practice increasingly views candidacy along a broader risk-adapted spectrum rather than a binary designation. This perspective may expand consideration of bladder preservation beyond only the most traditionally favorable subsets.

Appropriate workup includes exclusion of distant metastatic disease with cross-sectional imaging and careful assessment of local resectability through cystoscopic and radiographic evaluation. Treatment delivery considerations also influence outcomes. Whole-bladder RT remains the standard foundation of treatment, while pelvic nodal coverage may be individualized based on stage, nodal risk, and institutional practice. Advances in IMRT/IGRT and modern planning approaches have improved conformality and reduced practical toxicity barriers that historically limited treatment intensification or elective nodal irradiation in selected patients [14,15,19,20,21].

Importantly, many principles guiding candidate selection derive from prospective and institutional experience rather than rigidly validated criteria, and candidacy is best viewed as a risk-adapted multidisciplinary decision rather than a fixed checklist. Structured post-treatment surveillance remains integral to safe bladder preservation and should be considered part of the treatment strategy itself [22].

### 3.3. Toxicity, Quality of Life, and Functional Outcomes

Toxicity and functional outcomes are central to evaluating bladder preservation relative to radical cystectomy. Combined-modality therapy is associated with predictable acute genitourinary (GU) and gastrointestinal (GI) toxicities, including urinary frequency, dysuria, fatigue, and transient bowel symptoms, which are generally manageable and resolve after treatment completion [23]. Prospective trial data demonstrate acceptable rates of acute grade ≥ 3 toxicity, with most patients completing planned therapy without treatment-limiting adverse events [3,4,23].

Treatment discontinuation or failure to complete planned TMT appears relatively uncommon in prospective series, with most studies reporting treatment completion in the majority of patients and attrition generally below 10–20%, although rates vary by regimen intensity, age, and comorbidity [2,4,18].

Late toxicity following TMT is less common but remains clinically relevant. Reported late GU effects include hematuria, bladder fibrosis, and irritative urinary symptoms, while GI toxicity may manifest as proctitis or bowel dysfunction [24]. Long-term institutional series report low rates of severe late toxicity, supporting the safety of definitive CRT in appropriately selected patients [2,3,4,24]. Improvements in radiation delivery, including IMRT and image-guided techniques, have further reduced normal tissue exposure and likely contribute to favorable long-term tolerability [14,15,19]. Although secondary malignancy risk is a recognized long-term theoretical consideration following RT, the absolute risk appears low and is generally outweighed by the established oncologic benefits of bladder-preserving treatment.

Quality-of-life outcomes represent a principal rationale for organ preservation. Prospective assessments indicate that bladder-preserving therapy maintains urinary function and overall health-related quality of life in most patients, with recovery toward baseline functional status following treatment [23,24,25,26]. Comparative analyses between TMT and cystectomy cohorts demonstrate broadly similar global quality-of-life scores, with domain-level differences often favoring bladder preservation in urinary and sexual function [25,26]. These findings underscore the importance of incorporating patient-reported outcomes into shared decision-making.

Among complete responders, rates of durable bladder-intact survival remain high. Structured surveillance enables early detection of recurrence and timely salvage cystectomy when required, preserving oncologic safety while maintaining organ function in responders [2,3,4,7,22]. Taken together, contemporary toxicity and functional outcome data support bladder preservation as a clinically responsible organ-sparing strategy that balances disease control with long-term quality-of-life considerations.

Representative long-term outcomes from TMT studies are summarized in Table 2 and demonstrate durable oncologic control with favorable 5-year survival, modest salvage cystectomy rates, and preservation of bladder function in selected patients [2,3,7,11,18,23]. Available functional outcome data suggest bladder preservation can often be achieved without major compromise in urinary or bowel quality of life. While comparisons across studies are limited by heterogeneity in populations and endpoints, these data support TMT as an established organ-preserving strategy with durable oncologic and functional outcomes.

## 4. Bladder Preservation in Locally Advanced and Node-Positive Disease

While TMT was initially developed and validated primarily in patients with organ-confined MIBC, increasing attention has focused on the potential role of bladder preservation in patients with locally advanced and node-positive disease. Historically, these populations were often managed with systemic therapy alone per a metastatic paradigm, reflecting concerns regarding tumor burden, treatment response, and the risk of distant progression. However, advances in RT delivery and systemic therapy have prompted reconsideration of these boundaries [27,28,29,30].

Prospective and retrospective data support the feasibility of definitive RT-based strategies in node-positive MIBC. The phase II IMPART trial evaluated IMRT incorporating pelvic nodal irradiation for node-positive or high-risk node-negative bladder cancer, demonstrating acceptable toxicity and encouraging locoregional control outcomes [28]. These results underscore the capacity of modern RT techniques to safely treat extended target volumes while maintaining tolerability [19,28].

Emerging data support consideration of bladder preservation in carefully selected patients but do not establish it as a standard alternative to conventional management in this population. Patients most reasonably considered are those with limited nodal burden, favorable response to systemic therapy, medically inoperable disease, or those treated within experienced multidisciplinary programs or clinical trials. In contrast, more extensive nodal disease or bulky locally advanced presentations remain investigational contexts in which evidence remains limited.

Contemporary institutional series have also reported meaningful local control and survival outcomes among selected patients with node-positive MIBC treated with definitive CRT [29,30]. Importantly, improved systemic therapies in UC have strengthened the rationale for aggressive local management in this setting, as enhanced systemic disease control permits greater emphasis on durable locoregional treatment. Trials integrating immunotherapy with definitive RT have included patients with advanced local disease and have demonstrated promising safety and feasibility signals, although mature survival data remain pending [31,32,33].

Despite these encouraging findings, patterns-of-failure analyses consistently demonstrate that distant progression remains the predominant mode of relapse in locally advanced cohorts. This reality underscores the necessity of effective systemic therapy integration and careful patient selection when considering bladder preservation in higher-risk disease.

Overall, available evidence supports the feasibility of bladder preservation in selected patients with locally advanced or node-positive MIBC within experienced multidisciplinary settings. However, this application should be approached deliberately, with recognition of the elevated risk of distant failure and the ongoing need for prospective validation to clarify optimal sequencing and comparative effectiveness relative to surgical approaches.

## 5. Integration of Systemic Therapy with Bladder Preservation

### 5.1. Chemotherapy with CRT

Concurrent chemotherapy remains the most evidence-based intensification strategy in bladder-preserving CRT. BC2001 supports 5-fluorouracil/mitomycin C as an effective radiosensitizing regimen with durable benefit in locoregional control [3]. Cisplatin-based radiosensitization is commonly used in practice, but cisplatin ineligibility is frequent in MIBC due to renal dysfunction and comorbidity [16]. Biweekly or weekly gemcitabine has emerged as a practical radiosensitizer option supported by prospective randomized evidence comparing CRT regimens [17,18].

A key clinical reality is that systemic relapse remains a major cause of failure even after optimal local therapy, which is why modern bladder preservation increasingly needs to be discussed in parallel with the rapidly changing systemic therapy landscape.

### 5.2. Immunotherapy and Radiation

The integration of immune checkpoint inhibition (ICI) into bladder preservation is driven by both clinical precedent in advanced UC and biologic rationale for synergy with RT. Radiation can enhance tumor antigen presentation, increase immune cell infiltration, and promote immunogenic cell death, providing a mechanistic basis for combining RT with checkpoint blockade [31]. However, the extent to which these effects translate into clinically meaningful improvement beyond established TMT remains uncertain.

Early-phase studies evaluating ICI with definitive RT or CRT have demonstrated feasibility and acceptable toxicity in selected cohorts, with encouraging response rates [32]. These investigations establish that combining immunotherapy with curative-intent local therapy is operationally achievable, including in patients with advanced local disease or medical inoperability. The phase II DUART study, evaluating durvalumab with RT, provides representative evidence supporting the safety of this approach and has informed subsequent trial design [32].

Whether ICI meaningfully improves bladder-intact outcomes will depend on randomized evidence. The SWOG/NRG S1806 (INTACT) phase III trial is evaluating the addition of atezolizumab to standard CRT and is expected to clarify the incremental benefit of immunotherapy in the definitive setting [33]. Additional ongoing trials are examining alternative agents, sequencing strategies (concurrent versus maintenance), and biomarker-informed selection.

Several unresolved issues warrant caution. The magnitude of benefit over contemporary TMT is unknown, late GU toxicity requires mature follow-up, and the optimal patient population for immunotherapy intensification has not been defined. It is also unclear whether benefit will be concentrated in higher-risk subgroups or require biomarker enrichment. Until randomized results mature, ICI should be viewed as a promising but investigational strategy within bladder-preserving care.

### 5.3. Antibody Drug Conjugates with RT

Antibody–drug conjugates (ADCs) have emerged as a transformative therapeutic class in advanced UC, demonstrating meaningful survival benefit and prompting investigation into earlier disease settings. Enfortumab vedotin (EV), targeting nectin-4, and sacituzumab govitecan (SG), targeting Trop-2, have both shown improved outcomes in metastatic UC compared with standard chemotherapy [34,35]. More recently, the combination of EV with pembrolizumab (EV/P) demonstrated superior response rates and overall survival compared with platinum-based chemotherapy in the frontline metastatic setting, establishing EV/P as a new systemic standard of care in advanced UC [36]. Furthermore, most recently in EV-303/KEYNOTE 905 neoadjuvant EV/P demonstrated superior survival to neoadjuvant chemotherapy prior to cystectomy and has moved EV/P into the frontline, locazlied MIBC setting [37]. The magnitude and durability of systemic responses observed with EV/P have raised the question of whether highly active regimens might expand eligibility for bladder preservation by downstaging borderline locally advanced disease.

The biologic rationale for combining ADCs with RT is strong. ADC payload delivery induces tumor-selective cytotoxicity—often via DNA damage or microtubule disruption—which may complement radiation-induced cell kill. Preclinical studies suggest potential radiosensitization through augmentation of DNA damage, interference with cell-cycle progression, and modulation of the tumor microenvironment [38]. In contrast to conventional cytotoxic chemotherapy, ADCs offer targeted delivery, which may permit intensification of systemic therapy without proportionate increases in nonspecific toxicity.

Clinical interest in ADC–RT integration is therefore twofold. First, induction EV/P may achieve sufficient tumor regression to permit definitive bladder-preserving RT in patients who might otherwise be directed toward cystectomy. Second, concurrent or consolidative ADC strategies may enhance local control when combined with RT. Early-phase investigations evaluating EV in combination with RT for locally advanced UC have demonstrated feasibility and acceptable safety in preliminary cohorts, although long-term oncologic outcomes remain immature [39]. Trials such as CONSOLIDATE, STAR-EV, and EV-PRIME are actively evaluating the integration of EV/P with RT in locally advanced disease, with the goal of defining safety, tolerability, and bladder-intact disease control in a prospective setting [39,40,41].

Despite compelling rationale, important uncertainties remain. Optimal sequencing—induction followed by RT, fully concurrent delivery, or consolidation after CRT—has not been established. Potential overlapping toxicities, including dermatologic reactions, neuropathy, and hematologic suppression, warrant careful evaluation when combined with pelvic RT. Furthermore, it is not yet clear whether the impressive systemic efficacy of EV/P in metastatic disease will translate into improved bladder-intact survival in localized or regionally advanced UC. Randomized data will be required to determine whether ADC-based intensification improves outcomes beyond contemporary TMT or simply increases treatment-related morbidity.

At present, ADC–RT combinations represent a promising but investigational extension of bladder preservation. Their ultimate role will depend on demonstration of durable local control, acceptable late toxicity, and preservation of functional outcomes comparable to established approaches.

## 6. Patterns of Failure and Salvage After Bladder Preservation

Understanding patterns of recurrence following TMT is central to patient counseling, surveillance planning, and multidisciplinary management. Prospective trials and large institutional series consistently demonstrate that patients who achieve complete response after TMT often maintain durable local control, yet recurrence remains clinically relevant in a subset of patients. Failures may occur within the bladder, regional lymph nodes, or at distant sites, with distribution influenced by baseline tumor stage, completeness of TURBT, and systemic therapy integration [7,11].

Intravesical recurrence represents the most common local failure pattern. These events are most commonly non–muscle-invasive recurrences and are typically detected through structured cystoscopic surveillance [22]. Importantly, superficial recurrences can frequently be managed with endoscopic resection and intravesical therapy without loss of bladder preservation. In contrast, invasive local recurrence generally necessitates consideration of salvage cystectomy to maintain oncologic control.

Salvage cystectomy remains a critical safety component of bladder preservation strategies [11,42]. Cooperative group experiences and long-term institutional data demonstrate that delayed cystectomy for persistent or recurrent invasive disease can be performed with acceptable morbidity and favorable cancer-specific outcomes [7,42]. The feasibility of timely surgical intervention supports bladder preservation as a conditional strategy rather than an irreversible departure from cystectomy. Accordingly, early detection of incomplete response through rigorous post-treatment surveillance is essential [22].

Despite effective local management, distant metastatic progression remains a dominant mode of failure, particularly in patients with higher baseline stage or nodal involvement. This pattern underscores the continued importance of systemic therapy integration within bladder-preserving approaches [7]. Advances in imaging and circulating biomarkers such as circulating tumor DNA (ctDNA) have improved detection and management of metastatic relapse, and emerging data suggest that selected patients with limited metastatic burden may benefit from local consolidative therapy [43,44,45,46,47,48].

Taken together, recurrence patterns after TMT reinforce three principles: structured surveillance is mandatory; salvage surgery must remain readily accessible; and systemic disease control remains integral to long-term outcomes. Bladder preservation therefore requires coordinated multidisciplinary follow-up to maintain both oncologic safety and functional durability.

## 7. Management of Oligometastatic and Oligoprogressive Disease After Bladder Preservation

The recognition of oligometastatic and oligoprogressive disease states has influenced the management of recurrent UC following TMT. The oligometastatic state—characterized by a limited number of metastatic lesions amenable to definitive local therapy—represents an intermediate biological phenotype between localized and widely disseminated disease and provides a rationale for metastasis-directed therapy (MDT) [8,44]. Patterns-of-failure analyses after bladder preservation consistently demonstrate that distant relapse constitutes a dominant mode of progression, particularly in patients with higher-risk baseline features [7]. This distribution of failure raises the possibility that selected patients with limited metastatic burden may benefit from targeted local intervention.

MDT, most commonly delivered with stereotactic body radiation therapy (SBRT), has been increasingly applied in oligometastatic UC. Retrospective series and systematic reviews report high rates of local control with acceptable toxicity, supporting SBRT as a consolidative strategy in carefully selected patients [45,46]. In the context of prior bladder preservation, MDT may offer the opportunity to prolong disease control while maintaining bladder function and delaying escalation or change of systemic therapy. However, existing data remain largely retrospective and heterogeneous, with limited prospective validation specific to UC.

Oligoprogressive disease represents a related but distinct clinical scenario. Defined as progression at a limited number of sites during otherwise effective systemic therapy, oligoprogression provides a rationale for ablative treatment of resistant clones while preserving the benefit of ongoing systemic treatment [47]. This strategy has gained traction across multiple malignancies and is increasingly explored in UC, particularly in the era of highly active regimens such as EV/P and immune checkpoint inhibition. Improved functional imaging and structured surveillance have enhanced detection of limited progression, facilitating patient selection for MDT.

Despite biologic plausibility and encouraging institutional experiences, several uncertainties persist. Optimal patient selection criteria, timing relative to systemic therapy, and the impact of MDT on overall survival remain incompletely defined. Furthermore, it is unclear whether benefits reflect true modification of disease biology or selection of inherently favorable tumor phenotypes. Prospective trials—including basket and histology-specific studies—are necessary to clarify whether MDT meaningfully alters long-term outcomes in recurrent UC.

Incorporating MDT into post-bladder preservation care therefore requires careful multidisciplinary evaluation. When applied judiciously, MDT may extend disease control in selected patients; however, its role should remain individualized pending higher-level evidence.

## 8. Emerging Technologies and Future Directions

### 8.1. Radiation Technique Optimization

Advances in RT delivery have substantially refined bladder preservation and improved the therapeutic ratio of definitive treatment. Modern techniques—including IMRT and image guidance—permit improved target conformality and normal tissue sparing compared with historical approaches, reducing treatment-related toxicity while maintaining adequate tumor coverage [14,15,19]. These developments have facilitated safe delivery of definitive doses and expanded the feasibility of treating complex presentations, including patients requiring pelvic nodal irradiation or intensified local strategies.

Adaptive RT has emerged as a particularly relevant innovation in bladder treatment. Given marked inter- and intrafraction variability in bladder volume and position, adaptive workflows incorporating daily imaging with plan selection or reoptimization can improve target coverage while limiting dose to adjacent organs at risk [19,49,50]. Early clinical experiences demonstrate feasibility and favorable dosimetric outcomes, although prospective validation of clinical benefit remains ongoing. In parallel, growing evidence supports moderately hypofractionated regimens in bladder preservation, with comparable efficacy and acceptable toxicity relative to conventional fractionation [13]. These approaches offer logistical advantages and potential radiobiologic efficiency without compromising oncologic outcomes.

Rather than representing a paradigm shift, these technical advances reflect incremental improvements that enhance safety, expand eligibility, and support integration with systemic intensification strategies.

### 8.2. Response-Adapted and De-Escalation Strategies

Beyond technical optimization, contemporary investigation increasingly centers on treatment personalization. Clinical trials are evaluating response-adapted intensification or de-escalation strategies based on early response to CRT or systemic therapy, with the aim of preserving oncologic control while minimizing overtreatment [33]. Studies such as SWOG/NRG S1806 illustrate this approach by integrating novel systemic agents into established bladder-preserving treatment and evaluating their impact on response durability and survival [33].

Advances in imaging and molecular diagnostics may further refine risk stratification. Functional imaging and ctDNA assays are under investigation as tools to detect residual disease or early relapse, potentially enabling dynamic risk-adapted management [48]. These strategies raise the possibility of tailoring consolidation, surveillance intensity, or salvage timing according to molecular risk rather than relying solely on conventional imaging and cystoscopy.

However, many of these innovations remain investigational. Biomarker-guided adaptation must demonstrate reproducibility, clinical utility, and cost-effectiveness before routine adoption. Similarly, de-escalation strategies must preserve long-term bladder-intact survival, not simply short-term response metrics.

Future progress in bladder preservation will likely depend on integration of technological precision, biologic risk stratification, and disciplined prospective validation. The overarching goal remains consistent: maintain oncologic safety while maximizing functional preservation.

### 8.3. Molecular Profiling, Biomarkers and Immunohistochemical Evaluation

Increasing interest in precision oncology has prompted investigation into molecular tools that may refine patient selection, response prediction, and surveillance in bladder preservation. Transcriptomic and molecular subtype analyses suggest biologic heterogeneity within MIBC may influence radiosensitivity, treatment response, and risk of recurrence, raising the possibility that genomic classifiers could eventually help personalize bladder-preserving strategies [51]. Emerging urine-based and tissue-based assays are also being explored for treatment monitoring and post-treatment surveillance. For example, transcriptomic profiling approaches and evolving urinary biomarker platforms, including assays such as Bladder EpiCheck, may complement cystoscopic and cytologic surveillance in selected settings [52]. Although these approaches remain investigational and are not currently incorporated into routine selection for TMT, they represent a promising avenue for future biomarker-driven bladder preservation paradigms.

Immunohistochemical biomarkers may also contribute to future response-adapted bladder preservation strategies. In particular, interest in PD-L1 expression and related immune microenvironment markers reflects broader efforts to identify predictors of response to CRT and combined RT–immunotherapy approaches. While PD-L1 testing currently has no established routine role in selecting patients for TMT, these markers may have future relevance for biomarker-enriched trials incorporating ICI and other treatment intensification strategies. More broadly, integration of immunohistochemical and molecular profiling may help refine patient selection and therapeutic personalization, although prospective validation remains needed [53].

### 8.4. Patient-Derived Organoids and Precision Bladder Preservation

Patient-derived organoids (PDOs) represent an emerging translational platform with potential relevance to precision bladder preservation. By recapitulating aspects of tumor heterogeneity and therapeutic response ex vivo, PDO models may help inform treatment sensitivity, resistance mechanisms, and response-adaptive strategies in UC [54,55]. Particularly relevant to radiation oncology, these platforms may also support future study of radiosensitivity and biologically informed treatment intensification or de-escalation.

In the bladder preservation setting, PDOs may eventually contribute to refining patient selection or personalizing integration of CRT, immunotherapy, or emerging systemic agents. However, these applications remain investigational. Important limitations include incomplete modeling of the tumor microenvironment, challenges in clinical scalability, and lack of prospective validation supporting PDO-guided treatment selection. At present, PDOs should be viewed as a promising research platform rather than an established clinical decision tool [54,55].

## 9. Conclusions

Bladder preservation has evolved from a selective alternative to an established component of multidisciplinary management for appropriately selected patients with MIBC. Although definitive randomized comparisons are lacking, observational comparisons suggest favorable oncologic outcomes following TMT relative to surgical series, with potential for higher quality of life. These outcomes depend on careful patient selection, high-quality TURBT, appropriate systemic integration, and rigorous post-treatment surveillance.

Experience now suggests that bladder preservation may be feasible across a broader spectrum of disease presentations—including selected patients with locally advanced or node-positive disease—when delivered within coordinated multidisciplinary care. At the same time, patterns of failure after TMT reinforce that oncologic safety requires structured surveillance, timely salvage cystectomy, and continued attention to systemic disease control.

The therapeutic landscape in UC continues to evolve. The emergence of highly active systemic regimens—including ICI and ADC-based combinations such as EV/P—raises the possibility that improved systemic efficacy may expand candidacy for bladder preservation in borderline cases. Advances in RT delivery, adaptive planning, and MDT further refine local management strategies. However, intensification and personalization must be supported by prospective validation to ensure that gains in response translate into durable bladder-intact survival without compromising safety.

Bladder preservation is now a mature, evidence-based strategy that demands technical precision, multidisciplinary coordination, and disciplined follow-up. Its continued evolution will depend not simply on therapeutic innovation, but on rigorous clinical validation that preserves oncologic integrity while advancing organ preservation.

## 10. Literary Search Strategy and Methods

This narrative review was informed by literature identified through searches of PubMed/MEDLINE, Embase, and Google Scholar, focusing primarily on studies published from 2000 through 2025, with inclusion of seminal earlier studies where relevant. Search terms included combinations of “muscle-invasive bladder cancer,” “trimodality therapy,” “bladder preservation,” “chemoradiation,” “node-positive bladder cancer,” “immunotherapy,” and “antibody-drug conjugates.” Priority was given to landmark prospective trials, influential institutional series, clinical practice guidelines, and representative contemporary studies relevant to emerging bladder-preservation strategies. Additional references were identified through review of cited literature from key publications.

Generative AI (ChatGPT, OpenAI, GPT-5.3) was used solely for limited editorial support, including language editing and organizational refinement, and not for generation or interpretation of scientific conclusions.

## Figures and Tables

**Table 1:** Contemporary spectrum of candidate selection for bladder preservation with TMT, highlighting traditional ideal candidates, selected expanded indications requiring multidisciplinary consideration, and clinical scenarios generally associated with poor candidacy.

Ideal Candidates	Select Candidates (Multidisciplinary Consideration)	Generally Poor Candidates
Unifocal disease	Clinically node-positive disease	Multifocal disease
cT2–T3a tumors (<7 cm)	cT3b–T4a disease	Bulky tumor (>7 cm)
No CIS	Limited/focal CIS	Extensive CIS
Complete/maximal TURBT	Incomplete but substantial TURBT	Grossly incomplete TURBT
No or limited hydronephrosis	Unilateral hydronephrosis	Bilateral hydronephrosis
Good baseline bladder function		Poor bladder function
		Prior pelvic RT

**Table 2:** Long-term oncologic and functional outcomes from representative trimodality therapy (TMT) series and trials in muscle-invasive bladder cancer.

Study	Design/N	Population/Stage	Treatment Regimen	Median F/U	5-yr OS	5-yr DSS/CSS	Recurrence & Salvage Cystectomy	Functional/QoL Outcomes
[7] (RTOG pooled)	Pooled analysis; N = 468	cT2–T4a (61% cT2) N0M0	Induction CRT → consolidation CRT (cisplatin-based)	7.8 yr	57%	71%	5-yr muscle-invasive LF: 13% 5-yr non-muscle-invasive LF: 31% Overall cystectomy 21%; ~8% salvage	80% of 5-yr survivors retained intact bladder; low late pelvic toxicity in prior RTOG series
[2] (MGH)	Single-institution prospective series; N = 475	cT2–T4a (66% cT2) N0M0	Maximal TURBT + cisplatin-based CRT	7.2 yr	57% (cT2: 65%)	66% (cT2: 74%)	5-yr SC risk: 29%; declined to 16% in modern era 5-yr muscle-invasive LF: 16%	Bladder-intact DSS 52% (5-yr); favorable in contemporary era
[3,23]	Phase III RCT; CRT vs. RT; N = 360	cT2–T4a N0M0	64 Gy/32 fx or 55 Gy/20 fx ±5-FU + MMC	9.9 yr	48% (CRT) vs. 35% (RT) [3]	5-yr locoregional control: 63% (CRT) vs. 49% (RT) [23]	5-yr SC: 14% (CRT) vs. 22% (RT), *p* = 0.034 2-yr invasive recurrence: 18% (CRT) vs. 32% (RT)	No QoL detriment from CRT addition; RTOG G3/4 late toxicity 9.2% (CRT) vs. 17% (RT)
[18]	Phase II RCT; N = 66 eligible; 33 per arm	cT2–T4a (97% cT2)	5-FU/cis + BID RT (FCT) vs. gem + daily RT (GD)	5.1 yr	Not reported	3-yr DMF3: 78% (FCT) vs. 84% (GD)	3-yr BI-DMFS3: 67% (FCT) vs. 72% (GD) SC infrequent in both arms	Comparable late toxicity; both regimens feasible
[11]	Propensity-matched TMT vs. RC; IPTW N = 722; PSM N = 1119	cT2–T4a N0M0; TMT-eligible	Maximal TURBT + concurrent CRT	4.4–4.9 yr	73% (TMT) vs. 66% (RC) HR 0.70 *p* = 0.010	CSS: 84% (TMT) vs. 81% (RC) SHR 0.72 *p* = 0.071	SC in 13% of TMT patients MFS: 75% (TMT) vs. 74% (RC); NS	No prospective QoL comparison

Abbreviations: SC, salvage cystectomy; LF, local failure; DMFS, distant metastasis-free survival; MFS, metastasis-free survival; HR, hazard ratio; SHR, subdistribution hazard ratio.

## Data Availability

No new data were created or analyzed in this study. Data sharing is not applicable to this article.

## References

[1] Shipley W.U., Winter K.A., Kaufman D.S., Lee W.R., Heney N.M., Tester W.R., Donnelly B.J., Venner P.M., Perez C.A., Murray K.J. (1998). Phase III trial of neoadjuvant chemotherapy in patients with invasive bladder cancer treated with selective bladder preservation by combined radiation therapy and chemotherapy: Initial results of Radiation Therapy Oncology Group 89-03. J. Clin. Oncol..

[2] Giacalone N.J., Shipley W.U., Clayman R.H., Niemierko A., Drumm M., Heney N.M., Michaelson M.D., Lee R.J., Saylor P.J., Wszolek M.F. (2017). Long-term Outcomes After Bladder-preserving Tri-modality Therapy for Patients with Muscle-invasive Bladder Cancer: An Updated Analysis of the Massachusetts General Hospital Experience. Eur. Urol..

[3] James N.D., Hussain S.A., Hall E., Jenkins P., Tremlett J., Rawlings C., Crundwell M., Sizer B., Sreenivasan T., Hendron C. (2012). Radiotherapy with or without Chemotherapy in Muscle-Invasive Bladder Cancer. N. Engl. J. Med..

[4] Huddart R.A., Hall E., Hussain S.A., Jenkins P., Rawlings C., Tremlett J., Crundwell M., Adab F.A., Sheehan D., Syndikus I. (2013). Randomized Noninferiority Trial of Reduced High-Dose Volume Versus Standard Volume Radiation Therapy for Muscle-Invasive Bladder Cancer: Results of the BC2001 Trial (CRUK/01/004). Int. J. Radiat. Oncol..

[5] National Comprehensive Cancer Network NCCN Clinical Practice Guidelines in Oncology: Bladder Cancer. Version 2024. https://www.nccn.org.

[6] European Association of Urology EAU Guidelines on Muscle-Invasive and Metastatic Bladder Cancer. 2024 Edition. https://uroweb.org.

[7] Mak R.H., Hunt D., Shipley W.U., Efstathiou J.A., Tester W.J., Hagan M.P., Kaufman D.S., Heney N.M., Zietman A.L. (2014). Long-Term Outcomes in Patients with Muscle-Invasive Bladder Cancer After Selective Bladder-Preserving Combined-Modality Therapy: A Pooled Analysis of Radiation Therapy Oncology Group Protocols 8802, 8903, 9506, 9706, 9906, and 0233. J. Clin. Oncol..

[8] Hellman S., Weichselbaum R.R. (1995). Oligometastases. J. Clin. Oncol..

[9] Hoskin P.J., Rojas A.M., Bentzen S.M., Saunders M.I. (2010). Radiotherapy with Concurrent Carbogen and Nicotinamide in Bladder Carcinoma. J. Clin. Oncol..

[10] Song Y.P., Mistry H., Irlam J., Valentine H., Yang L., Lane B., West C., Choudhury A., Hoskin P.J. (2021). Long-Term Outcomes of Radical Radiation Therapy with Hypoxia Modification with Biomarker Discovery for Stratification: 10-Year Update of the BCON (Bladder Carbogen Nicotinamide) Phase 3 Randomized Trial (ISRCTN45938399). Int. J. Radiat. Oncol. Biol. Phys..

[11] Zlotta A.R., Ballas L.K., Niemierko A., Lajkosz K., Kuk C., Miranda G., Drumm M., Mari A., Thio E., Fleshner N.E. (2023). Radical cystectomy versus trimodality therapy for muscle-invasive bladder cancer: A multi-institutional propensity score matched and weighted analysis. Lancet Oncol..

[12] Efstathiou J.A., Spiegel D.Y., Shipley W.U., Heney N.M., Kaufman D.S., Niemierko A., Coen J.J., Skowronski R.Y., Paly J.J., McGovern F.J. (2012). Long-Term Outcomes of Selective Bladder Preservation by Combined-Modality Therapy for Invasive Bladder Cancer: The MGH Experience. Eur. Urol..

[13] Choudhury A., Porta N., Hall E., Song Y.P., Owen R., MacKay R., West C.M.L., Lewis R., A Hussain S., James N.D. (2021). Hypofractionated radiotherapy in locally advanced bladder cancer: An individual patient data meta-analysis of the BC2001 and BCON trials. Lancet Oncol..

[14] Lutkenhaus L.J., van Os R.M., Bel A., Hulshof M.C.C.M. (2016). Clinical results of conformal versus intensity-modulated radiotherapy using a focal simultaneous boost for muscle-invasive bladder cancer in elderly or medically unfit patients. Radiat. Oncol..

[15] Søndergaard J., Holmberg M., Jakobsen A.R., Agerbæk M., Muren L.P., Høyer M. (2014). A comparison of morbidity following conformal versus intensity-modulated radiotherapy for urinary bladder cancer. Acta Oncol..

[16] Rödel C., Grabenbauer G.G., Kühn R., Papadopoulos T., Dunst J., Meyer M., Schrott K.M., Sauer R. (2002). Combined-Modality Treatment and Selective Organ Preservation in Invasive Bladder Cancer: Long-Term Results. J. Clin. Oncol..

[17] Choudhury A., Swindell R., Logue J.P., Elliott P.A., Livsey J.E., Wise M., Symonds P., Wylie J.P., Ramani V., Sangar V. (2011). Phase II Study of Conformal Hypofractionated Radiotherapy with Concurrent Gemcitabine in Muscle-Invasive Bladder Cancer. J. Clin. Oncol..

[18] Coen J.J., Zhang P., Saylor P.J., Lee C.T., Wu C.-L., Parker W., Lautenschlaeger T., Zietman A.L., Efstathiou J.A., Jani A.B. (2019). Bladder Preservation with Twice-a-Day Radiation Plus Fluorouracil/Cisplatin or Once Daily Radiation Plus Gemcitabine for Muscle-Invasive Bladder Cancer: NRG/RTOG 0712—A Randomized Phase II Trial. J. Clin. Oncol..

[19] Huddart R., Hafeez S., Griffin C., Choudhury A., Foroudi F., Syndikus I., Hindson B., Webster A., McNair H., Birtle A. (2024). Dose-escalated Adaptive Radiotherapy for Bladder Cancer: Results of the Phase 2 RAIDER Randomised Controlled Trial. Eur. Urol..

[20] Patel S.A., Liu Y., Solanki A.A., Baumann B.C., Efstathiou J.A., Jani A.B., Chang A.J., Fischer-Valuck B., Royce T.J. (2023). Bladder only versus bladder plus pelvic lymph node chemoradiation for muscle-invasive bladder cancer. Urol. Oncol. Semin. Orig. Investig..

[21] Venkatesulu B., Liauw S.L., Joshi M., Baumann B.C., Yoo R., Roupret M., Choudhury A., Efstathiou J.A., Murthy V., Sargos P. (2022). Multidisciplinary Management and Radiotherapy Recommendations for Clinically and Pathologically Node-positive Bladder Cancer. Semin. Radiat. Oncol..

[22] Kassouf W., Aprikian A., Black P., Kulkarni G., Izawa J., Eapen L., Fairey A., So A., North S., Rendon R. (2016). Recommendations for the improvement of bladder cancer quality of care in Canada: A consensus document reviewed and endorsed by Bladder Cancer Canada (BCC), Canadian Urologic Oncology Group (CUOG), and Canadian Urological Association (CUA), December 2015. Can. Urol. Assoc. J..

[23] Hall E., Hussain S.A., Porta N., Lewis R., Crundwell M., Jenkins P., Rawlings C., Tremlett J., Sreenivasan T., Wallace J. (2022). Chemoradiotherapy in Muscle-invasive Bladder Cancer: 10-yr Follow-up of the Phase 3 Randomised Controlled BC2001 Trial. Eur. Urol..

[24] Zietman A.L., Sacco D., Skowronski U., Gomery P., Kaufman D.S., Clark J.A., Talcott J.A., Shipley W.U. (2003). Organ Conservation in Invasive Bladder Cancer by Transurethral Resection, Chemotherapy and Radiation: Results of a Urodynamic and Quality of Life Study on Long-term Survivors. J. Urol..

[25] Huddart R.A., Hall E., Lewis R., Porta N., Crundwell M., Jenkins P.J., Rawlings C., Tremlett J., Campani L., Hendron C. (2020). Patient-reported Quality of Life Outcomes in Patients Treated for Muscle-invasive Bladder Cancer with Radiotherapy ± Chemotherapy in the BC2001 Phase III Randomised Controlled Trial. Eur. Urol..

[26] Mak K.S., Smith A.B., Eidelman A., Clayman R., Niemierko A., Cheng J.-S., Matthews J., Drumm M.R., Nielsen M.E., Feldman A.S. (2016). Quality of Life in Long-term Survivors of Muscle-Invasive Bladder Cancer. Int. J. Radiat. Oncol..

[27] Rödel C., Weiss C., Sauer R. (2005). Organ preservation by combined modality treatment in bladder cancer: The European perspective. Semin. Radiat. Oncol..

[28] Hafeez S., McDonald F., Lalondrelle S., McNair H., Warren-Oseni K., Jones K., Harris V., Taylor H., Khoo V., Thomas K. (2017). Clinical Outcomes of Image Guided Adaptive Hypofractionated Weekly Radiation Therapy for Bladder Cancer in Patients Unsuitable for Radical Treatment. Int. J. Radiat. Oncol. Biol. Phys..

[29] Swinton M., Mariam N.B.G., Tan J.L., Murphy K., Elumalai T., Soni M., Ferrera A., Richardson C., Walshaw R., Mistry H. (2023). Bladder-Sparing Treatment with Radical Dose Radiotherapy Is an Effective Alternative to Radical Cystectomy in Patients with Clinically Node-Positive Nonmetastatic Bladder Cancer. J. Clin. Oncol..

[30] Haque W., Verma V., Butler E.B., Teh B.S. (2017). Chemotherapy Versus Chemoradiation for Node-Positive Bladder Cancer: Practice Patterns and Outcomes from the National Cancer Data Base. Bladder Cancer.

[31] Formenti S.C., Demaria S. (2013). Combining Radiotherapy and Cancer Immunotherapy: A Paradigm Shift. JNCI J. Natl. Cancer Inst..

[32] Joshi M., Tuanquin L., Zhu J., Walter V., Schell T., Kaag M., Kilari D., Liao J., Holder S.L., Emamekhoo H. (2023). Concurrent durvalumab and radiation therapy (DUART) followed by adjuvant durvalumab in patients with localized urothelial cancer of bladder: Results from phase II study, BTCRC-GU15-023. J. Immunother. Cancer.

[33] Singh P., Tangen C., Efstathiou J.A., Lerner S.P., Jhavar S.G., Hahn N.M., Costello B.A., Sridhar S.S., Du W., Meeks J.J. (2020). INTACT: Phase III randomized trial of concurrent chemoradiotherapy with or without atezolizumab in localized muscle invasive bladder cancer—SWOG/NRG1806. J. Clin. Oncol..

[34] Powles T., Rosenberg J.E., Sonpavde G.P., Loriot Y., Durán I., Lee J.-L., Matsubara N., Vulsteke C., Castellano D., Wu C. (2021). Enfortumab Vedotin in Previously Treated Advanced Urothelial Carcinoma. N. Engl. J. Med..

[35] Tagawa S.T., Balar A.V., Petrylak D.P., Kalebasty A.R., Loriot Y., Fléchon A., Jain R.K., Agarwal N., Bupathi M., Barthelemy P. (2021). TROPHY-U-01: A Phase II Open-Label Study of Sacituzumab Govitecan in Patients with Metastatic Urothelial Carcinoma Progressing After Platinum-Based Chemotherapy and Checkpoint Inhibitors. J. Clin. Oncol..

[36] Powles T., Valderrama B.P., Gupta S., Bedke J., Kikuchi E., Hoffman-Censits J., Iyer G., Vulsteke C., Park S.H., Shin S.J. (2024). Enfortumab Vedotin and Pembrolizumab in Untreated Advanced Urothelial Cancer. N. Engl. J. Med..

[37] Vulsteke C., Adra N., Danchaivijitr P., Sabadash M., Rodriguez-Vida A., Zhang Z., Atduev V., Göger Y.E., Rausch S., Kang S.-H. (2026). Perioperative Enfortumab Vedotin and Pembrolizumab in Bladder Cancer. N. Engl. J. Med..

[38] Natangelo S., Trapani D., Koukoutzeli C., Bielo L.B., Marvaso G., Jereczek-Fossa B.A., Curigliano G. (2024). Radiation therapy, tissue radiosensitization, and potential synergism in the era of novel antibody-drug conjugates. Crit. Rev. Oncol..

[39] Carriere P.P.J., Alhalabi O., Kamat A.M., Gao J., Goswami S., Campbell M.T., Shah A.Y., Jiang C.Y., Glover M., Bree K. (2026). CONSOLIDATE: A phase I/II study of radiotherapy combined with enfortumab vedotin (EV) for locally advanced bladder cancer with paired translational ctDNA and utDNA. J. Clin. Oncol..

[40] Zhang T., Woldu S.L., Lee M., Qin Q., Cole S., Arafat W., Jiang C., Wang J., Courtney K.D., DeVilbiss A. (2025). Neoadjuvant stereotactic radiotherapy and enfortumab vedotin: A phase I/II study for localized, cisplatin ineligible, muscle invasive bladder cancer (STAR-EV). J. Clin. Oncol..

[41] Koshkin V.S., Seyedin S.N., Zhang L., Curry N., Fitzgerald K.N., Shakhnazaryan N., Mitchell B., Kumar V., de Kouchkovsky I., Hong J.C. (2026). EV-PRIME: Phase Ib/II study of enfortumab vedotin and pembrolizumab combined with radiotherapy as a bladder-sparing trimodality therapy in muscle invasive bladder cancer. J. Clin. Oncol..

[42] Pieretti A., Krasnow R., Drumm M., Gusev A., Dahl D.M., McGovern F., Blute M.L., Shipley W.U., Efstathiou J.A., Feldman A.S. (2021). Complications and Outcomes of Salvage Cystectomy after Trimodality Therapy. J. Urol..

[43] Powles T., Kann A.G., Castellano D., Gross-Goupil M., Nishiyama H., Bracarda S., Jensen J.B., Makaroff L., Jiang S., Ku J.H. (2025). ctDNA-Guided Adjuvant Atezolizumab in Muscle-Invasive Bladder Cancer. N. Engl. J. Med..

[44] Weichselbaum R.R., Hellman S. (2011). Oligometastases revisited. Nat. Rev. Clin. Oncol..

[45] Franzese C., Francolini G., Nicosia L., Alongi F., Livi L., Scorsetti M. (2021). Stereotactic Body Radiation Therapy in the Management of Oligometastatic and Oligoprogressive Bladder Cancer and Other Urothelial Malignancies. Clin. Oncol..

[46] Angrisani A., Bosetti D.G., Vogl U.M., Castronovo F.M., Zilli T. (2024). Oligometastatic Urothelial Cancer and Stereotactic Body Radiotherapy: A Systematic Review and an Updated Insight of Current Evidence and Future Directions. Cancers.

[47] Palma D.A., Olson R., Harrow S., Gaede S., Louie A.V., Haasbeek C., Mulroy L., Lock M., Rodrigues P.G.B., Yaremko B.P. (2019). Stereotactic ablative radiotherapy versus standard of care palliative treatment in patients with oligometastatic cancers (SABR-COMET): A randomised, phase 2, open-label trial. Lancet.

[48] Christensen E., Birkenkamp-Demtröder K., Sethi H., Shchegrova S., Salari R., Nordentoft I., Wu H.-T., Knudsen M., Lamy P., Lindskrog S.V. (2019). Early Detection of Metastatic Relapse and Monitoring of Therapeutic Efficacy by Ultra-Deep Sequencing of Plasma Cell-Free DNA in Patients with Urothelial Bladder Carcinoma. J. Clin. Oncol..

[49] Vestergaard A., Muren L.P., Søndergaard J., Elstrøm U.V., Høyer M., Petersen J.B. (2013). Adaptive plan selection vs. re-optimisation in radiotherapy for bladder cancer: A dose accumulation comparison. Radiother. Oncol..

[50] Kong V., Hansen V., Hafeez S. (2021). Image-guided Adaptive Radiotherapy for Bladder Cancer. Clin. Oncol..

[51] Pepe P., Salemi M., Marchese G., Salluzzo M.G., Lanza G., Marino S., Schillaci F., Truda A., Pepe L., Pennisi M. (2024). Transcriptome Analysis in Patients with Muscle-invasive Bladder Cancer. In Vivo.

[52] Pepe L., Fiorentino V., Pizzimenti C., Riganati G., Franchina M., Micali M., Russotto F., Ieni A., Tuccari G., Fadda G. (2024). The Simultaneous Use of Bladder Epicheck^®^ and Urinary Cytology Can Improve the Sensitivity and Specificity of Diagnostic Follow-Up of Urothelial Lesions: Up-to-Date Data from a Multi-Institutional Cohort. Diseases.

[53] Germanà E., Pepe L., Pizzimenti C., Ballato M., Pierconti F., Tuccari G., Ieni A., Giuffrè G., Fadda G., Fiorentino V. (2024). Programmed Cell Death Ligand 1 (PD-L1) Immunohistochemical Expression in Advanced Urothelial Bladder Carcinoma: An Updated Review with Clinical and Pathological Implications. Int. J. Mol. Sci..

[54] Lee S.H., Hu W., Matulay J.T., Silva M.V., Owczarek T.B., Kim K., Chua C.W., Barlow L.M.J., Kandoth C., Williams A.B. (2018). Tumor Evolution and Drug Response in Patient-Derived Organoid Models of Bladder Cancer. Cell.

[55] Zhao H., Lin N., Ho V.W.S., Liu K., Chen X., Wu H., Chiu P.K., Huang L., Dantes Z., Wong K. (2025). Patient-Derived Bladder Cancer Organoids as a Valuable Tool for Understanding Tumor Biology and Developing Personalized Treatment. Adv. Sci..

